# Recent advances in phosphoproteomics based on mass spectrometry and its clinical application prospects

**DOI:** 10.3389/fphar.2026.1817319

**Published:** 2026-05-29

**Authors:** Jiuyu Gao, Ling Wang, Shengwei Shi, Zhihan Gu, Bofu Liu, Yu Cao

**Affiliations:** 1 Department of Emergency Medicine, Institute of Disaster Medicine and Institute of Emergency Medicine, West China Hospital, Sichuan University, Chengdu, China; 2 West China School of Medicine, Sichuan University, Chengdu, China; 3 Department of Emergency Medicine, West China Hospital, Sichuan University, Chengdu, China

**Keywords:** mass spectrometry, multi-omics integration, phosphoproteomics, precision medicine, single-cell analysis

## Abstract

Protein phosphorylation, a key post-translational modification, plays a crucial role in regulating cellular activities in eukaryotes. Dysregulation of phosphorylation is closely linked to the pathogenesis of various diseases Recent advancements in phosphoproteomics have driven substantial improvements in mass spectrometry technologies, enrichment strategies, and data analysis methodologies, thereby offering a powerful and precise tool for elucidating the spatiotemporal dynamics of phosphorylation events. Nevertheless, several challenges remain, such as the limited sensitivity for detecting low-abundance signals, throughput constraints of single-cell technologies, and the absence of standardized workflows, all of which impede clinical translation. Moving forward, AI-driven experimental design, spatial omics coupling, and interdisciplinary technological integration will drive the transition of phosphoproteomics from basic research to precision medicine, providing novel insights for disease diagnosis and treatment. This review aims to comprehensively summarize recent technological developments and future trends in phosphoproteomics, focusing on its clinical translation potential and associated challenges.

## Highlights

- Deep, high-throughput phosphoproteome coverage now routinely exceeds 30,000 sites within minutes, propelled by TIMS-DIA-PASEF and Orbitrap Astral platforms.
- Smart, stimuli-responsive enrichment materials and single-tube/µg-scale workflows enable robust phosphopeptide capture from scarce clinical or single-cell samples.
- AI-driven spectral libraries (directDIA, DIA-NN) and kinase-substrate prediction tools (SnapKin, KSTAR) convert massive phosphorylation data into actionable signalling networks.
- Multi-omics integration of phosphoproteomics with metabolomics or genomics pinpoints drug-resistance mechanisms and refines kinase-inhibitor stratification in cancer and neurodegeneration.
- Standardization, sensitivity for low-abundance organelle phosphoproteins, and large-scale clinical validation remain the key hurdles before routine precision-medicine deployment.

## 1 Introduction

Protein phosphorylation, one of the most prevalent and dynamically regulated post-translational modifications (PTMs), plays a critical role in modulating virtually all fundamental cellular processes in eukaryotes, including cell proliferation, metabolic regulation, signal transduction, and stress responses (Humphrey et al., 2015; Cohen, 2002). This biochemical process is intricately regulated by kinases and phosphatases, and its dysregulation is closely associated with the initiation and progression of major human diseases, including cancer, neurodegenerative disorders, and metabolic syndrome (Morshed et al., 2021; Muneer et al., 2024a). Consequently, the systematic analysis of the spatiotemporal dynamics of phosphorylation modifications is crucial not only for understanding the molecular mechanisms underlying biological processes but also for advancing precise disease diagnosis and treatment.

Understanding the spatiotemporal dynamics of phosphorylation through phosphoproteomics provides insights into cellular regulation and disease mechanisms. However, the clinical application of phosphoproteomics faces several challenges, such as limited sensitivity in detecting low-abundance phosphopeptides, throughput constraints of single-cell and spatial resolution technologies, the lack of large-scale validation of kinase-substrate interactions, and the absence of standardized workflows for clinical samples (Ctortecka et al., 2024; Polat and Özlü, 2014; Tsai et al., 2023).

In recent years, mass spectrometry (MS)-based phosphoproteomics has experienced revolutionary (TIMS), and high-resolution mass spectrometers enables the quantification of tens of thousands of phosphorylation sites in a single experiment, significantly improving coverage depth. Simultaneously, innovations in phosphopeptide enrichment strategies, from traditional metal affinity chromatography to smart responsive materials, have considerably enhanced the capture efficiency of low-abundance phosphopeptides (Xiao et al., 2023b; Giudice et al., 2023; Zhao Y. et al., 2022; Negroni et al., 2012).

This review aims to comprehensively summarize recent technological developments in phosphoproteomics, focusing on its clinical translation potential and associated challenges. The article will address four main areas: innovations in mass spectrometry technologies, phosphopeptide enrichment strategies, data analysis methods, and clinical application case studies. Key topics will include: technological breakthroughs that enable deep coverage of phosphorylation sites and high-precision quantification through novel acquisition modes and instrument platforms; solutions to analytical bottlenecks in single-cell and clinical sample analysis using smart materials and miniaturization technologies; the potential of multi-omics integration and artificial intelligence (AI) models in deciphering phosphorylation signaling networks; and strategies for enhancing the translational value of phosphoproteomic dynamic maps in cancer, neurodegenerative diseases, and metabolic disorders.

### 1.1 Innovations and optimizations in mass spectrometry technology

**FIGURE 1:**
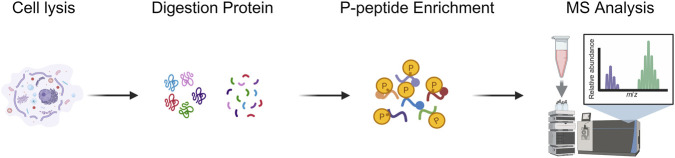
Overview of phosphoproteomics workflow. The process begins with cell lysis, followed by protein digestion to generate peptides. Phosphorylated peptides are then enriched, isolating the phosphoproteins. Finally, the samples undergo mass spectrometry (MS) analysis to identify and quantify the phosphopeptides, with the resulting data presented as mass-to-charge (m/z) ratios and relative abundance.

**FIGURE 2:**
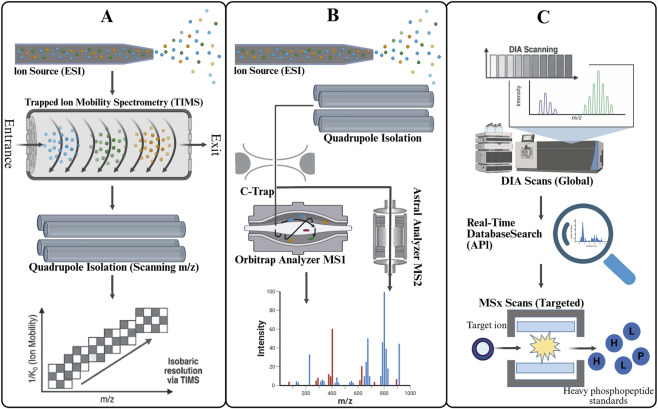
Schematic illustration of three advanced MS acquisition modes for phosphoproteomics. **(A)** DIA-PASEF on the timsTOF platform: Ions are separated by ion mobility (1/K_0_) in TIMS prior to quadrupole m/z isolation. DIA windows are arranged in a two-dimensional chessboard pattern across m/z and 1/K_0_ space, enabling high-throughput and isobaric resolution with short gradients. **(B)** Orbitrap Astral with DIA: Narrow 2 Th isolation windows are used for DIA. High-resolution MS1 scans (240k) are acquired in the Orbitrap, while MS2 scans are performed at 200 Hz in the Astral analyzer, achieving exceptional depth and pTyr sensitivity. **(C)** Hybrid-DIA: Global DIA scans are interleaved with real-time API-triggered targeted MSx scans. When a predefined heavy phosphopeptide standard is detected, the instrument switches to MSx mode to selectively isolate and fragment both the heavy standard and its endogenous light counterpart, achieving discovery and targeted verification in a single run. Created with BioRender.com.

Phosphoproteomics studies typically involve several key steps: (i) proteins are released from cells via cell lysis; (ii) proteins are digested by proteases to generate peptide mixtures; (iii) phosphopeptides are selectively captured through enrichment techniques to isolate low-abundance phosphopeptides; and finally, high-resolution mass spectrometry is used for precise identification and quantitative analysis of phosphorylation sites, as illustrated in Figure 1. Innovations in mass spectrometry constitute the primary driving force enabling the deep coverage and high-precision quantification of phosphoproteomics. Recent advancements in data acquisition modes, instrument resolution, and data analysis approaches have significantly improved the efficiency and reliability of phosphorylation site identification. The key features, performance metrics, and suitable applications of these representative platforms are summarized in Table 1 and Figure 2.

**TABLE 1:** Comparison of representative mass spectrometry platforms for high-throughput phosphoproteomics.

Platform	Acquisition mode	Sensitivity	Throughput	Coverage	Key features	References
Bruker timsTOF (DIA-PASEF)	DIA + TIMS	High (20 μg–1 μg)	Ultra-high (7–21 min)	∼13,000–35,000 sites	Speed & isobaric resolution via ion mobility	Skowronek et al. (2022), Oliinyk and Meier (2023), Oliinyk et al. (2023)
Orbitrap Astral (DIA)	DIA + Orbitrap/Astral	Very high (optimized for 30 μg)	Moderate-High (30 min-12 h)	∼30,000–81,000+ sites	Superior depth for pTyr & low-abundance sites	Lancaster et al. (2024)
Hybrid-DIA	DIA + targeted MSx	High (2.5 μg)	Moderate	Global + targeted panel	Discovery + targeted verification in one run	Bortel et al. (2024)
Optimized Automated Workflow (Orbitrap Exploris 480)	DDA/sequential enrichment	30 μg	30 min	∼16,000 sites	Standardized, reproducible enrichment	Petrosius et al. (2025)

This table compares four advanced MS platforms (Bruker timsTOF with DIA-PASEF, Orbitrap Astral with DIA, Hybrid-DIA, and the optimized automated workflow on Orbitrap Exploris 480) in terms of acquisition mode, typical sensitivity (sample input), throughput (LC gradient time), coverage (number of identified phosphosites), key advantages, limitations, recommended use-cases, and supporting references. The data illustrate the trade-offs between analysis speed, depth of coverage, and sample consumption, offering practical guidance for platform selection based on specific experimental objectives.

#### 1.1.1 Breakthroughs in novel mass spectrometry acquisition modes

##### 1.1.1.1 DIA-PASEF and trapped ion mobility spectrometry (TIMS)

The data-independent acquisition-parallel accumulation serial fragmentation (DIA-PASEF) technique, based on Trapped ion mobility spectrometry (TIMS), dynamically optimizes the m/z-ion mobility two-dimensional isolation window through algorithms, substantially improving the analysis of complex samples. The Bruker timsTOF platform, in conjunction with this technique, identified more than 35,000 high-confidence phosphorylation sites from 100 μg of EGF-stimulated HeLa cell lysates within just 21 min (Skowronek et al., 2022). Further integration of DIA-PASEF and TIMS with short-gradient liquid chromatography enabled the quantification of over 13,000 phosphopeptides from 20 μg of protein digests within only 7 min using ultra-short gradients, thereby successfully distinguishing isomeric phosphorylation sites that were unresolved by chromatography alone. The combination of DIA-PASEF and TIMS compensates for the separation loss in short gradients, enabling high-throughput, high-sensitivity phosphoproteomic analysis (Oliinyk and Meier, 2023).

Additionally, the high-throughput phosphoproteomic platform μPhos optimized sample handling processes, efficiently enriching phosphopeptides from protein samples as small as 1 μg. Combining the timsTOF Ultra and DIA-PASEF methods, the μPhos platform can quantify approximately 17,000 Class I phosphorylation sites from 20 μg samples. This platform has been successfully applied in leukemia cell drug response kinetics and mouse brain region spatial kinase activity analysis. Consequently, μPhos significantly outperforms traditional methods in terms of sensitivity, throughput, and reproducibility, offering a viable solution for large-scale perturbation experiments and trace sample research (Oliinyk et al., 2023).

##### 1.1.1.2 New high-sensitivity instrument platforms

The Orbitrap Astral mass spectrometer, combined with data-independent acquisition (DIA) technology using optimized chromatographic gradients and narrow isolation windows, enhances phosphopeptide analysis by improving resolution and scanning speed, enabling the detection of approximately 30,000 human phosphorylation sites within 30 min and 81,120 sites from a mouse multi-tissue map within 12 hours—offering five times the sensitivity of traditional methods and a tyrosine phosphorylation detection rate of 4.6% (Lancaster et al., 2024). Further systematic evaluation of the Orbitrap Astral platform for label-free single-cell and low-input proteomics has demonstrated enhanced proteome coverage from minimal sample inputs and provided optimized acquisition parameters for microscale phosphoproteomics (Petrosius et al., 2025). Furthermore, studies have optimized key parameters in the automated phosphopeptide enrichment process, such as bead-to-peptide ratios and ethylene glycol acid concentration, and combined sequential enrichment strategies to establish efficient methods for analyzing low sample volumes. The optimized workflow can identify 16,000 high-confidence phosphopeptides within 30 min from 30 μg of peptides using the Orbitrap Exploris 480 mass spectrometer, with a 20% increase in coverage using sequential enrichment strategies (Bortel et al., 2024). The timsTOF Ultra mass spectrometer, with upgraded ion sources and high-capacity Trapped ion mobility spectrometry (TIMS) technology, has greatly improved the sensitivity and depth of single-cell proteomic analysis. Under the same chromatographic conditions, it detects 15% more proteins than the previous generation timsTOF SCP. Its unique DIA-PASEF acquisition strategy and 25 Th wide isolation window allow for a dynamic range of four orders of magnitude in single-cell detection, successfully capturing more than 50 low-abundance E3 ubiquitin ligases. In high-throughput mode, the timsTOF Ultra still accurately identifies 1,200 proteomes (Ctortecka et al., 2024). Moreover, when combined with TIMS ion mobility separation and DIA-PASEF high-efficiency data acquisition, the timsTOF Ultra provides high-coverage, high-precision phosphoproteomic analysis, making it an indispensable tool for studying low-abundance modification sites in complex biological samples (Oliinyk and Meier, 2023).

##### 1.1.1.3 Hybrid data acquisition strategy (hybrid-DIA)

The Hybrid-DIA strategy combines data-independent acquisition (DIA) with targeted multi-stage tandem mass spectrometry (MSx) scanning, using phosphopeptide standards to trigger targeted scans of predefined endogenous peptides. Hybrid-DIA outperforms traditional DIA in detecting phosphorylation sites in low-input samples (2.5 μg), with quantitative performance comparable to the SureQuant targeted method, while retaining global coverage of the phosphoproteome. Hybrid-DIA effectively elucidated the differential phosphorylation dynamics between 3D spheroids and 2D cultured cells within DNA damage response and kinase signaling pathways. By integrating high-sensitivity targeted quantification with comprehensive proteomic discovery in a single workflow, it offers an efficient and robust solution for phosphoproteomic studies, particularly when working with limited sample amounts (Martínez-Val et al., 2023).

#### 1.1.2 Optimization of data acquisition strategies

The optimization of data acquisition strategies can be achieved through the integration of several advanced techniques: (i) the Hybrid Spectral Library and Prediction Library Strategy, (ii) Multidimensional Separation and Peak Capacity Enhancement, and (ii) Phosphorylation-Specific Fragmentation Technology. As illustrated in Table 2, these approaches work synergistically to improve the efficiency and accuracy of data acquisition, enabling more precise analysis and enhanced resolution of complex samples.

**TABLE 2:** Comparison of data acquisition optimization strategies for phosphoproteomics.

Strategy	Core principle	Key advantage	Key limitation	Representative tools/Methods	References
Hybrid Spectral Library	Combine DDA & DIA spectra	Reduces missing data; good for pTyr	Same type needs both DDA & DIA; more instrument time	Hybrid library from lung cancer lines	Kitata et al. (2021)
Prediction Library (Library-Free DIA)	AI extracts pseudo-DDA spectra from DIA	No DDA library needed; 1.5× more phosphosites	Reduced quantification stability for low-abundance peptides; higher false negatives	directDIA, DIA-NN	Wen et al. (2023)
Multidimensional Separation & Peak Capacity	Use ion mobility (TIMS) as orthogonal separation	Resolves isomeric phosphopeptides; ultra-fast 7-min gradients	Limited dynamic range; high computational demand	DIA-PASEF, TIMS	Skowronek et al. (2022), Oliinyk and Meier (2023)
Phosphorylation-Specific Fragmentation	ETD/ECD preserves labile phosphates	Precise site localization; avoids neutral loss	Low efficiency for small peptides; specialized instrument needed	ETD, ECD, EThcD	Daly et al. (2023), Muneer et al. (2024b)

This table summarizes four major strategies employed to enhance phosphopeptide identification and quantification: hybrid spectral libraries, AI-based prediction libraries (library-free DIA), multidimensional separation via ion mobility, and phosphorylation-specific fragmentation techniques. For each strategy, the core principle, key advantages, limitations, and representative tools are detailed, providing a practical reference for selecting appropriate approaches based on experimental requirements.

##### 1.1.2.1 The hybrid spectral library and prediction library strategy

The hybrid spectral library improves not only the identification of low-abundance phosphopeptides but also the precision of phosphorylation site localization. The integration of data-dependent acquisition (DDA) and data-independent acquisition (DIA) has significantly enhanced the accuracy and depth of phosphoproteomics analysis. In phosphoproteomics, a global analysis system that combines hybrid spectral libraries with DIA technology enables comprehensive quantitative analysis. A hybrid phosphopeptide spectral library, constructed from lung cancer cell lines and patient tissues, effectively revealed phosphorylation events associated with EGFR-TKI resistance and tumor progression, significantly reducing data missing rates across samples. It demonstrated particular advantages in detecting tyrosine phosphorylation sites, especially in the study of phosphorylation signal networks in low-abundance clinical samples (Kitata et al., 2021).

The use of AI-based prediction libraries, such as DIA-NN and directDIA technologies, has reduced dependency on traditional DDA spectral libraries. DirectDIA extracts pseudo-DDA spectra directly from DIA data and optimizes them using deep learning techniques. In both synthetic and real biological samples (e.g., Sequential Window Acquisition of All Theoretical Mass Spectra (SWATH) and DIA data), directDIA detected 1.5 times more phosphorylation sites than traditional DDA libraries, covering approximately 80% of the peptides in the DDA library. However, quantification stability for low-abundance peptides is significantly lower than in the DDA library. AI-based prediction libraries like DIA-NN show reduced sensitivity, particularly in synthetic samples, with a false-negative rate of up to 30%, primarily due to discrepancies between predicted fragment ions and experimental spectra. While AI prediction libraries and directDIA technologies still lag behind traditional DDA libraries in sensitivity and reliability, the latter, though limited in coverage, provides reliable results, particularly in site localization and false-positive rate control. Future research should focus on improving the accuracy of fragment ion predictions in AI models and enhancing the quantitative robustness of directDIA to strike a balance between sensitivity and reliability in high-throughput proteomic analysis (Wen et al., 2023).

##### 1.1.2.2 Multidimensional separation and peak capacity enhancement

Additionally, the combination of Trapped Ion Mobility Spectrometry (TIMS) and liquid chromatography, such as in DIA-PASEF technology, increases peak capacity and mitigates the co-elution of peptides in short gradients. For example, Oliinyk et al. quantified over 9,700 phosphopeptides using a 7-minute gradient, demonstrating high-efficiency separation capabilities under short gradients (Oliinyk and Meier, 2023). Phosphopeptide enrichment based on TiO_2_, coupled with high-performance liquid chromatography-mass spectrometry (LC-MS/MS), facilitates rapid phosphoproteomic analysis. This method refines the preprocessing workflow, making it more adaptable for high-throughput phosphorylation site identification in routine samples, such as cell lines (Carrera et al., 2021).

##### 1.1.2.3 Phosphorylation-specific fragmentation technology

Furthermore, Phosphorylation-Specific Fragmentation Technology, which differentiates sulfonated and phosphorylated peptides through neutral loss induced by low collision energy, is employed. Optimized Zr^4+^- immobilized metal affinity chromatography (IMAC) enrichment strategies and mass spectrometry-triggered methods based on higher-energy collisional dissociation (HCD) neutral loss triggered by low collision energy effectively distinguish sulfonated tyrosine (sY) from phosphorylated tyrosine (pY), reducing false-positive interference in traditional phosphopeptide enrichment (Daly et al., 2023). The phosphatase pre-treatment, combined with a multi-PTM analysis framework, has further optimized phosphorylation peptide identification strategies.

### 1.2 Phosphopeptide enrichment and sample preparation technologies

Phosphopeptide enrichment constitutes a pivotal step in phosphoproteomics research, as its efficiency and specificity directly influence the sensitivity, depth of coverage, and accuracy of mass spectrometry detection. In recent years, optimization of traditional enrichment methods and development of new materials have significantly enhanced the ability to capture low-abundance phosphopeptides, while the introduction of miniaturization and automation technologies has provided highly sensitive solutions for clinical sample analysis.

#### 1.2.1 Optimization and innovation of traditional enrichment methods

The optimization of traditional enrichment methods primarily involves improving the selectivity and efficiency of IMAC and Metal Oxide Affinity Chromatography (MOAC) techniques. IMAC achieves enrichment through electrostatic interactions between metal ions (such as Fe^3+^, Ti^4+^, Zr^4+^) and phosphate groups. Magnetic Fe^3+^-nitrilotriacetic acid (NTA) beads have replaced traditional column methods, maintaining high specificity while enabling high-throughput automated operations (Liu et al., 2022).

Further innovations have focused on functionalizing carbon-based nanomaterials—including hollow mesoporous carbon tubes (HMCT) and graphitic carbon nitride—with titanium, zirconium, or cerium ions. These functionalized materials exhibit enhanced loading capacity, excellent selectivity, and magnetic responsiveness, making them well-suited for the analysis of complex biological samples such as human serum and saliva (Liu et al., 2022; Brown et al., 2023; Wang et al., 2023).

Similarly, MOAC techniques rely on the chelation between metal oxides (e.g., TiO_2_, ZrO_2_) and phosphate oxygen. Recent developments include composite materials such as zeolite-loaded magnesium-aluminum-cerium ternary hydroxides (Zeolite@MAC), which have been systematically optimized for enrichment conditions (pH, adsorption time, material dosage) and successfully applied to capture cancer-related phosphorylated biomarkers in lung cancer metabolomics research (Yu et al., 2020). Additionally, anatase TiO_2_ nanoparticles prepared by sol-gel methods have demonstrated lowered detection limits and high sensitivity for direct quantification of low-abundance phosphopeptides in complex biological matrices (Wang and Ma, 2022). Table 3 provides a comparative summary of these diverse enrichment strategies, detailing their core materials, metal/oxide components, key advantages, sensitivity, and representative application cases.

**TABLE 3:** Comparison of phosphopeptide enrichment methods.

Type	Core material/Structure	Metal/Oxide	Key advantages	Sensitivity	Application cases	References
IMAC	Fe^3+^-NTA magnetic beads	Fe^3+^	High-throughput automation, reusable (20 cycles)	-	Phosphopeptide enrichment	Liu et al. (2022)
	CTs@DHA@Ti^4+^ (aqueous solvent synthesis)	Ti^4+^	High selectivity (BSA:β-casein = 2000:1), high loading capacity	2 fmol	Complex sample analysis (serum, saliva)	Sheng et al. (2024)
	G@C@Ti-Zr-HMCT (hollow mesoporous carbon tubes)	Ti^4+^/Zr^4+^	High loading capacity, excellent selectivity	0.2 fmol	Human serum phosphopeptide enrichment (Parkinson’s vs. healthy controls)	Hua et al. (2024)
	Fe_3_O_4_/g-C_3_N_4_-L-Ala-L-Gln-Ce^4+^	Ce^4+^	High surface area, hydrophilicity, positive charge, magnetic responsiveness	-	Phosphopeptide enrichment	Jiang et al. (2023)
MOAC	Zeolite@MAC (Mg-Al-Ce hydroxide composite)	Mg-Al-Ce	Ligand exchange and CePO_4_·xH_2_O inner-sphere complexation	-	Lung cancer serum phosphometabolite enrichment	Brown et al. (2023)
	Anatase TiO_2_ nanoparticles (sol-gel method)	TiO_2_	Ultra-low detection limit, high selectivity	0.24 pg/L	Quantitative analysis of casein hydrolysates in serum	Wang et al. (2023)

This table summarizes various enrichment strategies, including IMAC (Immobilized Metal Affinity Chromatography) and MOAC (Metal Oxide Affinity Chromatography), detailing their core materials/structures, metal/oxide components, key advantages, sensitivity, and application cases. The methods highlighted offer varying levels of sensitivity, loading capacity, and selectivity, with applications in complex sample analysis, disease biomarker enrichment, and quantitative studies.

In addition to optimization, innovations through chemical modifications and functionalized materials have been employed to enhance the targeting and enrichment capabilities of these methods. Chemical Ligation of Intact Phosphopeptides (CLIPP) specifically reacts with phosphate groups under Zn^2+^ catalysis to produce pyrophosphopeptides, which upon photolysis release intact phosphopeptides. Then, it innovatively employs lanthanide metal ions to efficiently hydrolyze pyrophosphate bonds under mild conditions, thereby demonstrating a novel and efficient catalytic mechanism. CLIPP demonstrates complementary advantages to traditional IMAC/MOAC methods: avoiding strong acid/base treatment, compatible with unstable modification analysis, offering a gentle and highly selective new strategy for phosphorylation site identification in complex samples, particularly suitable for tyrosine phosphorylation and potentially fragile modification studies (Brown et al., 2023).

Furthermore, titanium ion-rich hydrazide-linked porous organic polymers (hydrazide-POPs-Ti^4+^) synthesized by a two-step method integrate hydrophilic interaction chromatography (HILIC) and immobilized metal ion affinity chromatography (IMAC) dual mechanisms, enabling efficient simultaneous enrichment of glycopeptides and phosphopeptides (Wang et al., 2023).

#### 1.2.2 Development of novel smart responsive materials

In recent years, smart responsive materials have received attention in phosphopeptide enrichment due to their dynamic regulation capabilities. They can be classified according to stimulus source:

Internal stimuli (pH, enzymes, redox, etc.): Such as pH-responsive supramolecular polymers that dynamically bind phosphopeptides through acid-base regulation (Yu et al., 2020).

External stimuli (light, temperature, etc.): Temperature-responsive materials (such as poly-N-isopropylacrylamide, PNIPAm) utilize lower critical solution temperature (LCST) phase transitions to reversibly capture phosphopeptides (Wang and Ma, 2022); Light-responsive materials (such as spiropyran-modified magnetic nanoparticles) enhance selectivity through photo-controlled isomerization (SP↔MC/MCH^+^) (Zhao et al., 2021).

Additionally, triple-responsive materials (MNP-pSPA-b-pNIPAm) integrate light, pH, and temperature response mechanisms to achieve dynamic enrichment and gentle release of phosphopeptides (Zhao et al., 2023). Compared to traditional materials, their advantages include: avoiding strong acid elution, protecting phosphorylation site integrity; enhancing selectivity through multiple responses; and reusability.

#### 1.2.3 Breakthroughs in low sample volume preparation techniques

Breakthroughs in low sample volume preparation techniques, including workflow improvements and efficient processing of clinical samples, provide a breakthrough guarantee for phosphoproteomics in low sample volume preparation. For example,,the SOP-Phos single-tube workflow consolidates sample processing steps (lysis, digestion, and TiO_2_ enrichment) into a single tube, quantifying approximately 6,500 phosphopeptides from 2,500 cells with missing values <1% (Muneer et al., 2024a). Tandem tip technology (such as C18-IMAC-C18) could identify 3,000–9,500 phosphopeptides from 1 to 10 μg protein through multi-step chromatography column tandem (Tsai et al., 2023). The application of automated microplatforms, such as proteoCHIP EVO 96 coupled with Evosep One liquid chromatography system and timsTOF Ultra mass spectrometer, enables single-cell proteome analysis without manual operation (Ctortecka et al., 2024). In addition, phosphoproteomics enrichment technologies face technical bottlenecks such as non-specific adsorption, sample loss, and masking of low-abundance phosphopeptides, requiring the development of highly selective materials to improve efficiency. Currently, the combination of material chemistry innovation with miniaturization and automation technologies has significantly improved detection sensitivity and clinical translation potential, with efficient and reproducible enrichment methods being the key support for transforming biomarkers into diagnostic kits (Zittlau et al., 2023; Urban, 2022). SARS-CoV-2 virus particles were purified by ultracentrifugation, effectively removing fetal bovine serum proteins from the culture medium. Subsequently, Phos-tag magnetic beads were used to enrich phosphorylated proteins from virus particles, and LC-MS/MS identified the Ser79-specific phosphorylation site of NP. This workflow reduced interference from non-viral phosphorylation signals by combining physical purification with selective enrichment of phosphorylated proteins (Ino et al., 2022). Moreover, an efficient nanoscale phosphoproteomics workflow based on tandem tips integrates surfactant-assisted one-pot preparation (SOP), tandem C18-IMAC-C18 enrichment technology, and improved isobaric labeling signal enhancement strategy (iBASIL). This method significantly reduces sample loss and improves processing throughput by reducing sample transfer steps and non-specific adsorption, providing a feasible solution for single-cell and spatially resolved phosphoproteomics (Tsai et al., 2023).

### 1.3 Data analysis and computational methods

The exponential growth in data generated by mass spectrometry technologies has introduced significant challenges in phosphoproteomics data analysis, including high complexity, noise interference, and difficulties in functional annotation. Recent advances in deep learning, network modeling, and multi-omics integration have substantially enhanced the ability to interpret phosphorylation site functions.

#### 1.3.1 Phosphorylation site identification and functional annotation

Phosphorylation site identification and functional annotation involve two key aspects: optimizing site localization algorithms to accurately determine the positions of phosphorylation sites and performing functional annotation and inferring kinase activity to better understand the biological significance of these sites, the relevant prediction websites are summarized in Table 4. Tools such as PhosR and Perseus integrate localization probability scores to filter phosphorylation sites, retaining high-confidence sites to reduce false positives (Xiao et al., 2023a). Mass spectrometry fragmentation analysis reveals that the sulfate group in sulfonated tyrosine (sY) undergoes neutral loss at low collision energy, while phosphorylated tyrosine (pY) requires higher energy to release the phosphate group. This difference allows for selective differentiation of sY from pY via energy-dependent neutral loss, avoiding the complex multi-stage fragmentation workflow and achieving high-confidence site identification in a single MS/MS analysis (Daly et al., 2023). For phosphorylation isomers of adjacent serine/threonine residues, the key distinction lies in the fragment ion information with complete sequence coverage and modification retention, as acquired through high-precision mass spectrometry. For instance, electron transfer dissociation (ETD) or electron capture dissociation (ECD) techniques preferentially cleave peptide N-Cα bonds while preserving labile phosphate groups, generating c and z• product ions (cf. b and y ions from HCD). The resulting fragment ion series provide improved sequence coverage of the modified region. Site localization is further enhanced through probability model calculations and fragment ion matching scores when combined with statistical tools and machine learning algorithms (Muneer et al., 2024b). Phosphorylation site functional annotation often relies on kinase-substrate association analysis using external databases. Examples include PhosphoSitePlus (Hornbeck et al., 2015) and iPTMnet (Huang et al., 2018), which integrate experimentally validated phosphorylation sites and kinase regulatory information. Additionally, Phospho. ELM provides a specialized repository of phosphorylation sites occurring within functional linear motifs (e.g., SH2, SH3, PDZ domains) across 12 eukaryotic species, complementing broader PTM databases with motif-centric annotations (Dinkel et al., 2011). piNET is a comprehensive network platform that integrates multiple data sources, such as UniProt, PhosphoSitePlus, iPTMnet, SIGNOR, and deep learning prediction tools (DeepPhos), to facilitate efficient annotation of proteomics data, PTM network analysis, and mechanism interpretation (Shamsaei et al., 2020). Additionally, the machine learning-based computational tool, pHisPred, offers precise identification of histidine phosphorylation (pHis) sites, significantly outperforming existing tools and being the first to predict eukaryotic pHis sites effectively (Zhao J. et al., 2022). The Kinase-Substrate Transfer to Activity Relationships (KSTAR) algorithm, based on graph theory and statistics, reconstructs kinase-substrate networks through heuristic pruning, substrate enrichment assessment, and kinase activity scoring (using the Mann-Whitney U test). It does not require quantitative phosphorylation data, overcoming challenges such as data sparsity and experimental bias. Experiments have shown that KSTAR’s prediction accuracy for tyrosine kinases is 50% higher than existing methods, with a data loss tolerance exceeding 60%, enabling precise detection of dynamic kinase activity, such as those in EGFR/HER2(40).

**TABLE 4:** Databases and computational tools for phosphorylation site prediction and kinase-substrate analysis.

Tool	Type	Brief description	Website/Availability	References
PhosphoSitePlus	Database	Curated repository of >547k human phosphorylation sites, kinase-substrate pairs, disease associations	www.phosphosite.org	Hornbeck et al. (2015)
Phospho.ELM	Database	328k eukaryotic phosphosites with domain-specific annotations (SH3, PDZ, etc.)	Phospho.elm.eu.org	Dinkel et al. (2011)
iPTMnet	Database	Integrated PTM knowledge graph from UniProt, PhosphoSitePlus, and literature mining	Research.bioinformatics.udel.edu/iptmnet/	Huang et al. (2018)
piNET	Web platform	Proteomics data annotation, PAM network analysis, integrates DeepPhos predictions	–	Shamsaei et al. (2020)
DeepPhos	Prediction tool	Multi-CNN deep learning for S/T/Y phosphorylation site prediction across species	Github.com/pcm820/DeepPhos	Shamsaei et al. (2020)
pHisPred	Prediction tool	First tool for eukaryotic histidine phosphorylation site identification	Github.com/xiaofengsong/pHisPred	Zhao J et al. (2022)
SnapKin	Prediction tool	Deep learning ensemble for kinase-substrate prediction; 50% higher pTyr accuracy	Github.com/PYangLab/SnapKin	Xiao et al. (2023b)
KSTAR	Network tool	Kinase activity inference from phosphoproteomics; tolerates >60% data loss	Github.com/NaegleLab/KSTAR	Sam et al. (2022)
SELPHI2.0	Prediction tool	ML-based kinase-substrate interaction; extends to understudied kinases	Selphi2.0 webserver	Maier et al. (2025)
INKA	Network tool	Kinase activity scoring, network visualization, pathway enrichment	–	Mantini et al. (2021)

This table provides an overview of widely used resources for phosphoproteomics data interpretation, including curated databases (PhosphoSitePlus, Phospho.ELM, iPTMnet), integrated web platforms (piNET), and specialized prediction tools (DeepPhos, pHisPred, SnapKin, KSTAR, SELPHI2.0, INKA). For each resource, the type, core function, key features, and availability are indicated, offering a consolidated reference for functional annotation and network analysis of phosphorylation sites.

#### 1.3.2 Kinase-substrate relationship prediction and network modeling

Deep learning-based methods for predicting kinase-substrate relationships systematically analyze protein phosphorylation patterns (Varshney and Mishra, 2023). These methods use prediction results to reconstruct and dynamically model phosphorylation signaling networks, unveiling the structural characteristics of kinase-substrate interaction networks and their dynamic regulatory mechanisms in signal transduction. SnapShot deep learning ensemble for Kinase-substrate prediction (SnapKin) addresses the issue of verified substrate scarcity in phosphoproteomics data by integrating false positive generation and data resampling strategies. This approach significantly improved the model’s predictive performance in kinase perturbation data (Xiao et al., 2023b). More recently, SELPHI2.0—a machine learning framework that predicts kinase-substrate interactions at the phosphosite level—has demonstrated improved performance compared to existing methods and extends predictions to understudied kinases. The model is integrated into a web server to facilitate unbiased analysis of phosphoproteomics data (Maier et al., 2025). By integrating BioID proximity labeling, kinase perturbation, and phosphorylation motif matching, a systematic identification of endogenous kinase substrates has been achieved, offering a robust framework for analyzing kinase-substrate relationships (Niinae et al., 2021).

PhuEGO extracts functional modules from global phosphoproteomics data using three-layer network propagation and ego-network decomposition. In SARS-CoV2 infection datasets, phuEGO identifies active signal networks related to viral replication, uncovering connections between complex signaling networks (Giudice et al., 2023). The integration of phosphoproteomics and metabolomics data, combined with PCA analysis, revealed the dynamics of tyrosine phosphorylation signaling networks in EGF-stimulated MCF10A cells (Hillis et al., 2024).

#### 1.3.3 Challenges and critical considerations in AI-Driven phosphoproteomics analysis

While AI-based tools have revolutionized phosphoproteomics data interpretation, several critical limitations and sources of bias must be carefully considered to avoid over-interpretation of results.

Library-Free DIA Analysis Tools (e.g., DIA-NN, directDIA). Although deep learning-based spectral library prediction reduces reliance on experimentally derived DDA libraries, studies have shown that quantification stability for low-abundance phosphopeptides remains significantly lower than that achieved with project-specific DDA libraries (Kitata et al., 2021). In synthetic peptide benchmarks, DIA-NN exhibited higher false-negative rates compared to library-based approaches, raising concerns about its sensitivity for detecting biologically critical but low-stoichiometry phosphorylation events (Kitata et al., 2021). Furthermore, the performance of these tools can vary depending on the complexity of the sample and the specific phosphopeptide enrichment strategy employed, underscoring the need for careful parameter optimization and validation (Hua et al., 2024).

Kinase-Substrate Prediction Tools (e.g., SnapKin, KSTAR). Tools such as SnapKin and KSTAR have demonstrated impressive accuracy in reconstructing kinase signaling networks from phosphoproteomics data (Xiao et al., 2023b; Niinae et al., 2021). However, their predictive power is inherently constrained by the completeness and bias of the underlying training databases. The majority of experimentally validated kinase-substrate relationships are derived from a relatively small subset of well-studied kinases (e.g., EGFR, MAPK, CDK families), leading to a database bias that favors the detection of canonical signaling pathways while potentially overlooking the activity of under-characterized or tissue-specific kinases (Shamsaei et al., 2020). Additionally, the interpretability of deep learning ensemble models like SnapKin remains a challenge; while they provide high-confidence predictions, elucidating the specific sequence or structural features driving these predictions is non-trivial (Xiao et al., 2023b). KSTAR, while robust to missing data (>60% tolerance), relies on statistical pruning that may eliminate true but weakly supported kinase-substrate edges in small sample cohorts (Niinae et al., 2021). Therefore, predictions from these tools should be complemented with orthogonal validation, such as *in vitro* kinase assays or genetic perturbation experiments, before drawing definitive biological conclusions (Hillis et al., 2024).

Benchmarking and Reproducibility. A recent systematic benchmarking of commonly used DIA software suites for phosphoproteomics revealed substantial variability in the number of identified phosphosites and their quantitative precision across different tools and parameter settings (Hua et al., 2024). This highlights the urgent need for community-adopted gold-standard reference datasets and standardized benchmarking protocols to objectively evaluate and compare the performance of emerging AI-based methods in phosphoproteomics.

#### 1.3.4 Multi-omics integration and systems biology research

Phosphoproteomics-metabolomics analysis has provided new insights into the mechanisms of cancer metabolic reprogramming. For example, EGFR/PI3Kα inhibitor screening of signal-dependent phosphorylation events, multi-omics integration, Kyoto Encyclopedia of Genes and Genomes (KEGG) pathway enrichment analysis, and the use of an inducible CRISPR interference (CRISPRi) system to validate the conditional knockdown of metabolic enzymes and wild-type/phospho-mutant complementation were employed (Hillis et al., 2024). Thus, a joint analysis of phosphoproteomics and metabolomics was conducted to systematically investigate the regulatory network of tyrosine phosphorylation on metabolic enzymes.

Furthermore, in lung cancer, abnormal accumulation of phosphorylated metabolites is linked to the overactivation of glycolysis and mitochondrial oxidative phosphorylation pathways. Overexpression of enzymes such as hexokinase, phosphofructokinase, and lactate dehydrogenase drives increased glycolytic flux, which supports lung cancer cell proliferation and survival (Batool et al., 2023).

By integrating lung cancer phosphoproteomics, proteomics, and genomics data, and using phosphorylation pathway databases such as KEGG and PTMSigDB alongside the INKA analysis tool, associations between driving mutations and phosphorylation signaling networks can be examined. For example, DIA phosphorylation quantification revealed significant enrichment of ERK1/2 kinase-specific motif (S/TP) substrates in EGFR T790M-resistant cells, with increased phosphorylation at downstream sites such as ERK1-T202/Y204. Post-translational modification signature enrichment analysis (PTM-SEA) and INKA scoring validated the resistance mechanism driven by sustained MAPK pathway activation (Mantini et al., 2021).

#### 1.3.5 Data preprocessing and reproducibility challenges in clinical phosphoproteomics

Despite advances in acquisition and identification algorithms, the translation of phosphoproteomics to clinical settings is critically hindered by challenges in data preprocessing and cross-study reproducibility. These issues are particularly acute for large-scale cohort studies where batch effects, missing values, and platform variability can obscure true biological signals.

##### 1.3.5.1 Missing value imputation

Phosphoproteomics data are characterized by a high proportion of missing values, often exceeding 20%–30% in DIA experiments. Critically, these missing values are frequently Missing Not At Random (MNAR), arising from biological factors such as low stoichiometry phosphorylation or stochastic precursor selection in DDA mode, rather than random technical noise (Mantini et al., 2021). Standard imputation methods (e.g., k-nearest neighbors, global mean) may introduce bias if the MNAR nature is not accounted for. Specialized approaches, such as left-censored imputation or PhosR’s structure-aware algorithms, have been developed to better handle phosphoproteomics-specific missingness patterns (Kim et al., 2021).

##### 1.3.5.2 Batch effect correction

Clinical phosphoproteomics inevitably involves sample processing across multiple days, enrichment batches, or liquid chromatography-mass spectrometry (LC-MS) runs, introducing systematic non-biological variation known as batch effects. Uncorrected batch effects can severely confound differential phosphorylation analysis, particularly when case and control samples are unevenly distributed across batches. Tools originally developed for genomics and transcriptomics, such as ComBat (empirical Bayes framework) and its phosphoproteomics-adapted variants, are now routinely applied to mitigate these effects (Dubois et al., 2022). However, careful experimental design—including randomized sample allocation and the inclusion of common reference samples (e.g., mixed tissue lysates or peptide pools) in each batch—remains the gold standard for minimizing batch-related artifacts.

##### 1.3.5.3 Normalization strategies

Unlike global proteomics where total protein amount serves as a stable reference, phosphoproteomics normalization is complicated by the fact that global phosphorylation levels can shift dramatically under perturbation (e.g., kinase inhibitor treatment). Common normalization approaches include median/mean centering, quantile normalization, or normalization to spiked-in internal standards (e.g., heavy-labeled phosphopeptide standards). A recent benchmarking study revealed that the choice of normalization method can significantly affect the number and identity of differentially regulated phosphosites, underscoring the need for careful method selection and transparent reporting (Dubois et al., 2022).

##### 1.3.5.4 Cross-platform reproducibility

The comparability of phosphoproteomics data generated across different instrument platforms (e.g., timsTOF vs. Orbitrap Astral) or acquisition modes (DDA vs. DIA) remains a significant barrier to multi-center clinical studies. Systematic evaluations have demonstrated that while the most abundant phosphosites are consistently identified across platforms, the overlap of low-abundance sites can drop below 50% due to differences in ion sampling and fragmentation efficiency (Colomé et al., 2022). The adoption of standardized reference materials (e.g., well-characterized cell line digests) and community-wide quality control metrics (e.g., Proteomics Quality Control Consortium guidelines) is essential for establishing robust, reproducible phosphoproteomics workflows suitable for regulatory-grade clinical applications (Hua et al., 2024; Niemi and Pagliarini, 2021).

### 1.4 Clinical application prospects

**FIGURE 3:**
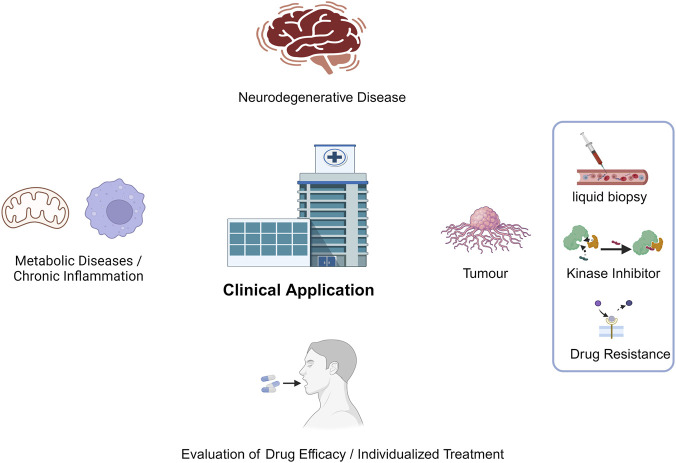
Clinical applications of phosphoproteomics. The diagram illustrates various clinical applications of advanced diagnostic techniques and treatments, including the management of neurodegenerative diseases, metabolic disorders, chronic inflammation, and tumors. It highlights the role of liquid biopsy for monitoring drug efficacy, drug resistance, and personalized treatments, such as kinase inhibitors, to address individual patient needs. Additionally, the figure emphasizes the application of phosphoproteomics in these clinical areas to better understand disease mechanisms and optimize therapeutic strategies.

The potential of phosphoproteomics in disease mechanism interpretation, biomarker discovery, and precision medicine is gradually being realized in clinical practice. Phosphoproteomics offers new insights for early diagnosis, efficacy evaluation, and drug development in cancer, neurodegenerative diseases, and metabolic diseases, as illustrated in Figure 3.

#### 1.4.1 Cancer precision diagnosis and treatment

Cancer precision diagnosis and treatment involves the discovery of biomarkers, the application of liquid biopsy techniques, and the development of kinase inhibitors. Additionally, understanding resistance mechanisms and exploring combination therapies are crucial for improving therapeutic outcomes. In lung cancer, the detection of EGFR tyrosine phosphorylation sites revealed resistance mechanisms involving interactions between multiple signaling pathways, such as Hippo-EGFR-ERBB in EGFR-TKI resistant cells, indicating their potential as biomarkers for predicting targeted therapy resistance (Muneer et al., 2024a). Kinase activity levels of ERBB2, as inferred from phosphoproteomics data, partially correlate with clinical HER2 status in breast cancer patients, providing potential insights for predicting sensitivity to HER2-targeted therapy (Sam et al., 2022). Phosphorylated proteins in extracellular vesicles (EVs) demonstrate significant clinical potential in liquid biopsies. For instance, specific phosphorylated proteins, such as Ral GTPase activating protein subunit α-2, in plasma EVs from breast cancer patients can serve as diagnostic markers (Chen et al., 2017). Moreover, phosphoproteomics analysis of urinary EVs has substantial potential in cancer signaling pathway research and early cancer diagnosis (Wu et al., 2018). In EGFR-TKI resistant lung cancer cells, reduced phosphorylation levels at the S127 site of YAP, a key regulator of the Hippo pathway, lead to its nuclear translocation and activation of downstream target genes (e.g., EGFR/ERBB family receptors). Simultaneously, enhanced MOB1A-T35 phosphorylation and RASSF1A-S131 phosphorylation events form a positive regulatory network with EGFR/ERBB signaling, driving resistance (Martínez-Val et al., 2023). Cyclin-Dependent Kinase (CDK) inhibitors, screened based on phosphorylation profiles, significantly inhibit tumor growth in KRAS-mutated pancreatic cancer models (Kazi et al., 2021). Additionally, phosphoproteomics analysis can reveal dynamic changes in tumor cell signaling pathways, including resistance mechanisms after kinase inhibitor treatment (e.g., lapatinib), where compensatory activation of other kinases, such as mesenchymal-epithelial transition factor (MET), may be reflected in phosphorylated proteins in EVs (Lee et al., 2013).

#### 1.4.2 Early diagnosis of neurodegenerative diseases

Tandem mass spectrometry (tMS)-based methods can specifically detect Tyr682-phosphorylated APP protein peptide segments in blood monocytes, distinguishing them from non-phosphorylated peptide segments. Expanding sample sizes and including healthy control populations to establish reference value ranges may help solidify this approach as a clinical early diagnostic marker (Reveglia et al., 2021). Phosphoproteomics analysis of serum from Parkinson’s disease (PD) patients reveals that abnormal aggregation of α-synuclein inhibits vesicle transport between the endoplasmic reticulum and Golgi apparatus, while the abnormal accumulation of PINK1 protein on the mitochondrial membrane is associated with mitochondrial dysfunction. These findings highlight the crucial role of phosphorylated proteins in regulating organelle function in PD pathogenesis, offering new insights for pathological classification (Hua et al., 2024). In Alzheimer’s disease (AD) brain tissues, quantitative levels of tau protein phosphorylation sites (e.g., pS579) significantly correlate with Tau pathology severity, with pT217 identified as an AD-specific biomarker. Cluster analysis revealed that phosphorylation signals in the “Tau” module are highly correlated with AD diagnosis (Morshed et al., 2021).

#### 1.4.3 Metabolic diseases and chronic inflammation regulation

By systematically integrating authoritative public phosphoproteomics repositories including PhosphoSitePlus and MitoCarta (Niemi and Pagliarini, 2021), Niemi.et al., established a comprehensive phosphorylation landscape of mammalian mitochondrial proteins. Through combined phosphoproteomic profiling and metabolic phenotyping of genetic knockout models, we elucidated the functional mechanisms of endogenous mitochondrial phosphatases and their substrate-specific regulatory networks (Xiao-yin et al., 2022). Furthermore, the investigation identified phosphorylation-dependent activation of NF-κB signaling components (particularly p65 Ser536 phosphorylation) in alveolar macrophages as a critical molecular determinant of inflammatory exacerbation, providing mechanistic insights for refining anti-inflammatory therapeutic strategies.

#### 1.4.4 Drug efficacy evaluation and personalized treatment

Phosphoproteomics is rapidly transitioning from basic research to clinical practice, with its core value in revealing the dynamic regulation of disease-specific signaling networks and providing actionable molecular targets for precision medicine. In the future, phosphorylation analysis is expected to become a routine method for early screening, efficacy monitoring, and personalized treatment of major diseases such as cancer and neurodegenerative diseases. In EGFR inhibitor treatment, real-time detection of ERK/MAPK pathway phosphorylation levels (e.g., pERK Thr202/Tyr204) can evaluate drug targeting efficiency (Zittlau et al., 2023). Machine learning models, based on phosphorylation signaling networks such as KSTAR, analyze kinase activity, assisting in the stratified treatment of breast cancer patients (Sam et al., 2022). The miniaturization of single-cell phosphoproteomics and spatial resolution technologies facilitates rare sample analysis. Phosphorylation-genomics-metabolomics joint databases, such as TCPA and CPTAC, are constructing multi-omics integration platforms that foster cross-dimensional biomarker discovery (Mantini et al., 2021).

## 2 Disscussion

Despite significant advances in phosphoproteomics technology and its applications, several challenges remain in its clinical translation. First, there is inadequate sensitivity for detecting low-abundance phosphopeptides in complex samples, such as mitochondrial phosphoproteins (Thongboonkerd and Chaiyarit, 2022). Second, the complexity of the data and issues with reproducibility present substantial barriers to the clinical application of phosphoproteomics. Poor cross-laboratory data comparability arises from batch effects and variability in analytical workflows (Lou et al., 2023). A recent systematic benchmarking of informatics workflows for DIA-based single-cell proteomics has provided comprehensive recommendations for data preprocessing—including sparsity reduction, missing value imputation, normalization, and batch effect correction—offering a valuable framework for standardizing phosphoproteomics analyses (Wang et al., 2025). Additionally, the functional annotation of kinase-substrate networks urgently requires innovative approaches, combining deep learning and data augmentation strategies. Many biomarkers remain inadequately validated in large-scale cohorts, underscoring the critical need for multi-center collaboration to integrate phosphorylation data, genomic information, and clinical outcomes (Di et al., 2023). AI-driven experimental design can reduce trial-and-error costs, while dynamic network modeling tools are essential for analyzing the spatiotemporal evolution of phosphorylation signals, offering novel strategies to counteract resistance mechanisms. The integration of multi-dimensional technologies is a key development direction. In therapeutic strategies, phosphorylation dependency and phosphorylation vaccines show considerable potential for precision treatment.

We believe that the deep integration of materials science, artificial intelligence, and clinical medicine,as well as breakthroughs in Frontier tools such as single-cell technologies and spatial omics—will rapidly transform basic clinic research into precision-medicine applications, offering entirely new intervention strategies for major diseases like cancer and neurodegenerative disorders.

## Glossary

AD: Alzheimer’s Disease
AI: Artificial Intelligence
CDK: Cyclin-Dependent Kinase
CID: Collision-Induced Dissociation
CLIPP: Chemical Ligation of Intact Phosphopeptides
CRISPRi: CRISPR interference
CTs: Chitosan
DDA: Data-Dependent Acquisition
DIA: Data-Independent Acquisition
DIA-NN: Data-Independent Acquisition by Neural Networks
ECD: Electron Capture Dissociation
EGFR: Epidermal Growth Factor Receptor
ERBB: Erythroblastic Oncogene B
ERK: Extracellular Signal-Regulated Kinase
ETD: Electron Transfer Dissociation
EV: Extracellular Vesicle
HCD: Higher-energy Collisional Dissociation
HILIC: Hydrophilic Interaction Liquid Chromatography
HMCT: Hollow Mesoporous Carbon Tube
IMAC: Immobilized Metal Ion Affinity Chromatography
INKA: Inference of Kinase Activity
KEGG: Kyoto Encyclopedia of Genes and Genomes
LC-MS/MS: Liquid Chromatography-mass Spectrometry
LCST: Lower Critical Solution Temperature
MAPK: Mitogen-Activated Protein Kinase
MET: Mesenchymal-Epithelial Transition factor
MOAC: Metal Oxide Affinity Chromatography
MS: Mass Spectrometry
MSx: Targeted Multi-stage Tandem Mass Spectrometry
NTA: Nitrilotriacetic Acid
PASEF: Parallel Accumulation–Serial Fragmentation
PD: Parkinson’s Disease
PTM: Post-Translational Modification
PTM-SEA: Post-Translational Modification Signature Enrichment Analysis
pHis: Phosphohistidine
pY: Phosphotyrosine
sY: Sulfated Tyrosine
SOP: Surfactant-One-Pot
SWATH: Sequential Window Acquisition of All Theoretical Mass Spectra
TKI: Tyrosine Kinase Inhibitor
TIMS: Trapped Ion Mobility Spectrometry
